# Abundance of G-Quadruplex Forming Sequences in the Hepatitis Delta Virus Genomes

**DOI:** 10.1021/acsomega.3c09288

**Published:** 2024-01-09

**Authors:** Václav Brázda, Natália Valková, Michaela Dobrovolná, Jean-Louis Mergny

**Affiliations:** † Institute of Biophysics of the Czech Academy of Sciences, Královopolská 135, Brno 621 00, Czech Republic; ‡ Faculty of Chemistry, Brno University of Technology, Purkyňova 118, Brno 61200, Czech Republic; § Laboratoire d’Optique et Biosciences, Ecole Polytechnique, CNRS, INSERM, Institut Polytechnique de Paris, Palaiseau 91120, France

## Abstract

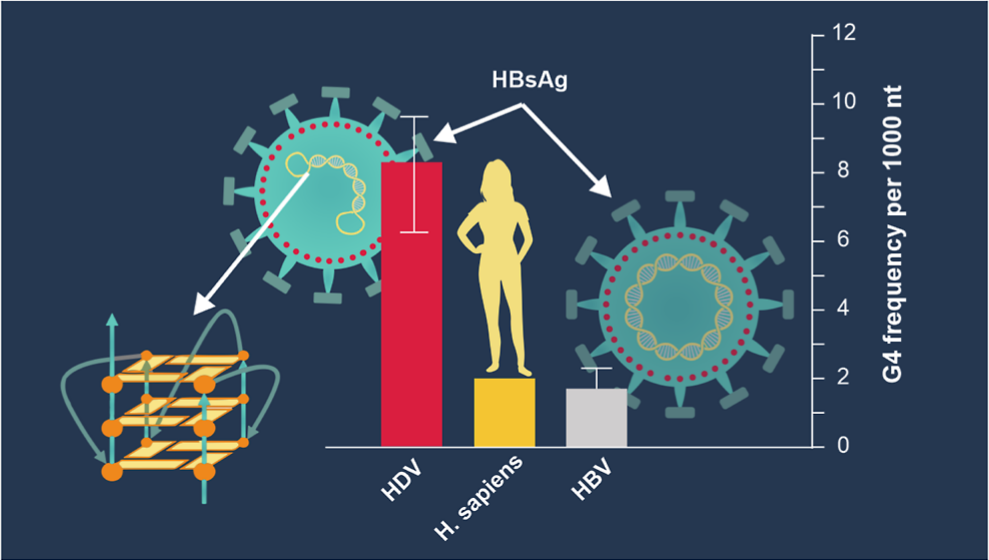

Hepatitis delta virus (HDV) is a highly unusual RNA satellite virus that depends on the presence of hepatitis B virus (HBV) to be infectious. Its compact and variable single-stranded RNA genome consists of eight major genotypes distributed unevenly across different continents. The significance of noncanonical secondary structures such as G-quadruplexes (G4s) is increasingly recognized at the DNA and RNA levels, particularly for transcription, replication, and translation. G4s are formed from guanine-rich sequences and have been identified in the vast majority of viral, eukaryotic, and prokaryotic genomes. In this study, we analyzed the G4 propensity of HDV genomes by using G4Hunter. Unlike HBV, which has a G4 density similar to that of the human genome, HDV displays a significantly higher number of potential quadruplex-forming sequences (PQS), with a density more than four times greater than that of the human genome. This finding suggests a critical role for G4s in HDV, especially given that the PQS regions are conserved across HDV genotypes. Furthermore, the prevalence of G4-forming sequences may represent a promising target for therapeutic interventions to control HDV replication.

## Introduction

Hepatitis delta virus (HDV) is an unusual RNA virus; it is considered a satellite virus or subviral agent, as it can propagate only in the presence of hepatitis B virus (HBV), which provides viral proteins necessary for its replication and assembly. Due to underdiagnosis, it is estimated that 12 to 72 million people are currently affected by this virus. Considered the most severe form of viral hepatitis, HDV infection in individuals with chronic hepatitis B (known as superinfection) is associated with significant complications. HDV coinfection can lead to a more severe form of liver disease compared to HBV infection alone. The coinfection of HDV with HBV or superinfection in chronic carriers of HBV can result in fulminant hepatitis,^1^ cirrhosis, and an increased risk of hepatocellular carcinoma (HCC).^2^ When combined with HBV, hepatitis D exhibits the highest mortality rate among all other hepatitis infections, at 20%.

The HDV genome consists of a small circular single-stranded RNA molecule. This gRNA has a length of approximately 1.7 kb and exists in a rod-like structure due to extensive base pairing within the RNA molecule.^3,4^ The HDV genome contains a single protein-coding region, which encodes the HDAg delta antigen, ribozyme sequences that cleave the viral genome and antigenome during replication, and regulatory elements.^5^ The HDV genome has negative polarity and is a template for transcription of HDV mRNA, allowing the production of HDAg. The gRNA also serves as a template for the synthesis of antigenomic RNA using the rolling-circle strategy. Antigenomic RNA then acts as a template for the replication of gRNA again by using the rolling-circle strategy. Because it encodes only HDAg, HDV uses host cell DNA-dependent RNA polymerases to replicate and assemble new viral particles.^6^ It also hijacks HBsAg, the HBV envelope glycoprotein, for its own virions. HDV exhibits considerable genetic variability, and currently, eight major HDV genotypes (HDV-1 to HDV-8) have been characterized.^7^ These genotypes show distinct geographic distributions and are associated with different clinical outcomes and disease progression.^8^

The G-quadruplex (G4) structure is a DNA or RNA secondary structure formed by sequences rich in guanine (G) bases. It consists of stacked G-tetrads, where four guanines interact through Hoogsteen hydrogen bonding, creating a stable planar structure. RNA G4s are key motifs of the transcriptome.^9^ G4s were identified in many viruses, including important human pathogens,^10,11^ and are considered to be promising therapeutic targets.^12,13^ Therefore, we analyzed the presence and localization of potential G-quadruplex-forming sequences (PQS) in the HDV genome. We found that PQS in HDV are numerous, with a density more than four times higher than that of HBV and the human genome. This suggests an important role for G4s in HDV, especially given that the tracks with the potential to form G4s are well conserved.

## Materials and Methods

### Genomes

474 accessible HDV genomes were downloaded from the Comprehensive Hepatitis D Virus Database (HDVdb)^14^ and classified according to the HDVdb records into different groups. In the G4Hunter prediction analysis, genotype categorization was used together with classification based on the geographical region of HDV origin. The genome groups included HDV-1 (322 genomes), HDV-2 (19 genomes), HDV-3 (11 genomes), HDV-4 (38 genomes), HDV-5 (17 genomes), HDV-6 (16 genomes), HDV-7 (45 genomes), and HDV-8 (6 genomes). Geographical region groups included Asia (186 genomes), Africa (230 genomes), Europe (24 genomes), North (3 genomes), and South America (9 genomes). The NC_076103.1 genome (genotype 3) is assigned in the NCBI database as the reference HDV genome.^15^

### G4Hunter Analyses

All HDV genomes were analyzed using G4Hunter^16^ to identify PQS. The analysis parameters were set for a window size of 25 nucleotides and a variable threshold of 1.2 or higher. The presence of PQS was searched for detection thresholds above 1.2, 1.5, 1.8, and 2.0. PQS appearing at higher thresholds have a higher propensity to fold into G4s and form very stable structures.^17^ The raw data for all genomes analyzed are in Supporting Information 01, which also provides information on NCBI accession numbers, length, GC count, percentage GC genome content, and the number and frequency of PQS.

Of note, G4Hunter does not define which guanines are involved in G4 formation—this is often not trivial when dealing with irregular repeats, and G4 structures are often full of surprises. It is therefore often difficult to assess parameters such as loop length when G-tracks are poorly defined; only high-resolution structures would allow a reliable assessment. Nevertheless, the motifs with the highest G4Hunter scores tend to correspond to quadruplexes with relatively short loops and at least three quartets.

### Construction of LOGO Sequences

Multiple sequence alignment was performed in UGENE using the MUSCLE algorithm with default parameters. All sequences for ancient and modern HBV genomes were uploaded into UGENE software,^18^ and locations of PQS sequences were extracted. The LOGO sequence was generated in aligned sequences and the WebLogo 3 tool.^19^

### Statistical Evaluation

**Figure 1:**
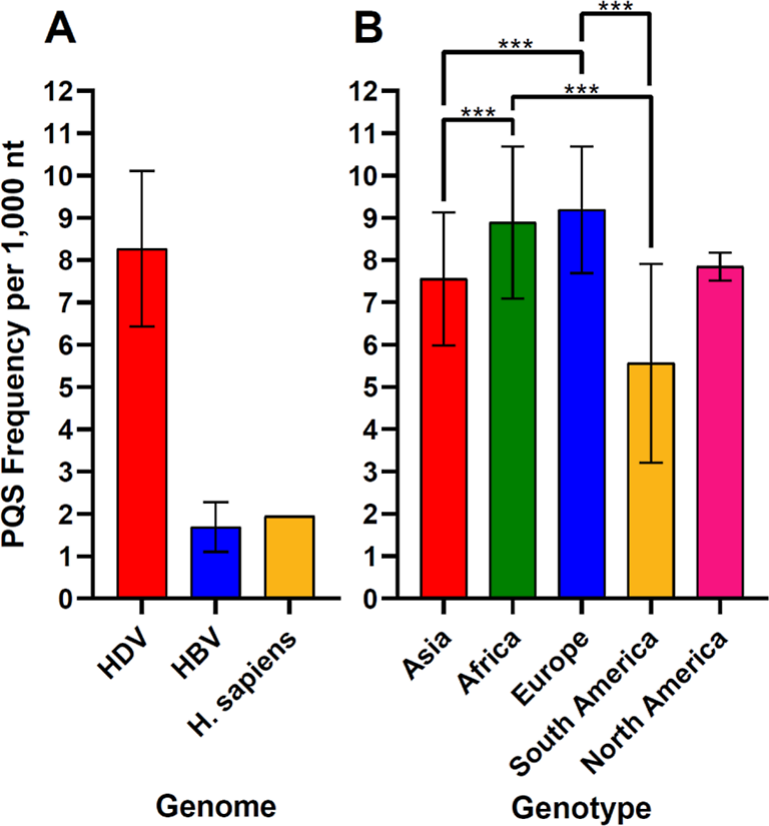
Comparison of PQS frequencies (A) in the HDV, HBV, and human genomes; (B) in HDV genomes isolated in different continents. Significant differences are shown by asterisks: *p-value < 0.05; **p-value < 0.01; ***p-value < 0.001.

**Figure 2:**
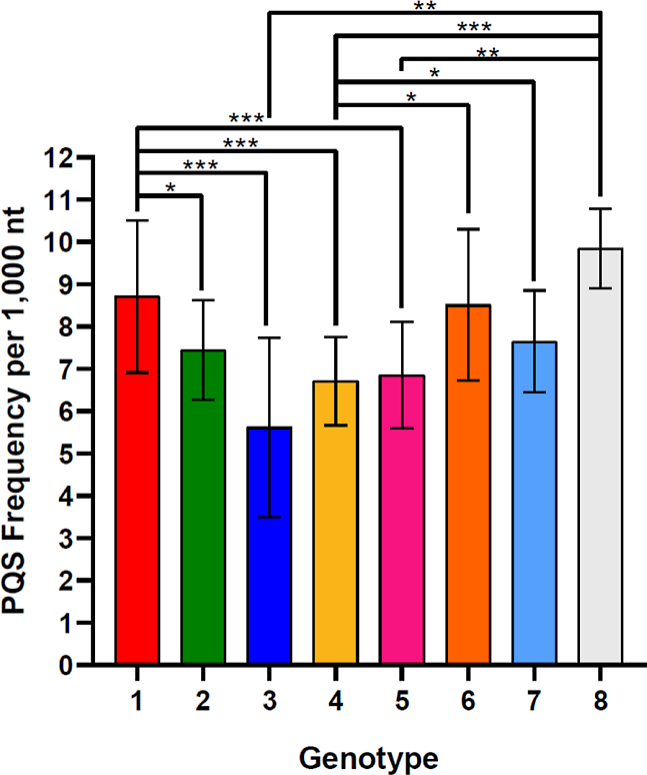
Comparison of the PQS frequencies in HDV genotypes. Significant differences are shown by asterisks: *p-value < 0.05; **p-value < 0.01; ***p-value < 0.001.

Data with G4Hunter results were merged in an Excel file for statistical evaluation. The G4Hunter results, lengths, and GC content of the sequences analyzed are accessible in Supporting Information 01. Bar charts (Figures 1 and 2) were generated in GraphPad Prism (v 8.0.1). The Dunn post hoc test with Bonferroni correction was employed to test the statistical significance of the difference between pairs of groups, the significant differences are shown in Figures 1 and 2, and all statistical data are available in Supporting Information 01.

## Results and Discussion

A total of 474 accessible HDV genomes were downloaded from HDVdb,^14^ and the presence of PQS was analyzed using G4Hunter. HDV genomes were of relatively similar length, ranging from 1533 to 1755 nucleotides. Using a G4Hunter threshold 1.2, we found a total number of 6675 PQSs and an average GC content of 58.6%. On average, a PQS frequency relative to the genome GC content of 14.07 (PQS/GC %) was observed. In contrast to the PQS frequency of 1.69 in HBV and 1.94 in the human genome,^20^ a significantly higher PQS frequency of 8.25 per 1000 nucleotides was found for the HDV virus (Figure 1A). We then compared the PQS in the negative and positive strands. Seven sequences were identified on the positive strand, while five were identified on the negative strand within the reference genome (NC_076103.1). Across all genomes examined, 48% of all PQSs were detected on the negative strand, while 52% were located on the positive strand.

However, the PQS frequencies among individual HDV genomes varied considerably between 2.35 and 13.65 PQS per 1000 nt. The count of all sequences analyzed across distinct regions and genotype categories, together with information on median genome length, shortest, and longest genome lengths, as well as mean, minimum, and maximum observed PQS frequency, are shown in Table 1. Comparison of genomes shows strong variation among individual HDV genomes. The HF679405 genome had the lowest PQS count with 4 sequences for a 1699 nt long genome, giving a PQS frequency of 2.35. In contrast, the KY463678 genome contains the largest number of PQS, with 23 candidate sequences, corresponding to a frequency of 13.7 PQS per 1000 nt. For the G4Hunter prediction analysis, we used the classification based on the geographical region of HDV origin. Comparisons of HDV genomes isolated from various continents showed that sequences with lower PQS frequencies were predominantly identified in South America, followed by Asian and North American genomes, contrary to genomes with higher PQS frequencies primarily in HDV genomes of African and European origin (Figure 1B).

**Table 1:** Data are for G4Hunter Analyses of HDV Viruses^a^

groups	seq *n*	length nt	GC %	PQS *n*	mean PQS	min PQS	max PQS	PQS per GC
all HDV	474	1705	58.6	6675	8.25	2.35	13.65	14.07
Continent								
Asia	186	1700	58.5	2390	7.55	2.92	12.28	12.92
Africa	230	1706	58.8	3467	8.88	5.23	13.66	15.12
Europe	24	1705	58.7	376	9.19	6.46	12.30	15.64
North America	3	1700	57.9	40	7.84	7.65	8.23	13.53
South America	9	1700	59.9	85	5.56	2.35	9.40	9.27
Genotype								
1	322	1705	58.3	4775	8.68	2.92	13.66	14.85
2	19	1707	56.3	241	7.07	5.28	9.96	11.93
3	11	1700	60.0	105	5.62	2.35	9.40	9.36
4	38	1709	59.3	436	6.71	4.68	8.78	11.33
5	17	1717	59.2	200	6.86	5.23	9.93	11.59
6	16	1714	57.8	233	8.51	5.27	11.67	14.71
7	45	1698	59.1	584	7.65	5.30	9.99	12.95
8	6	1711	59.5	101	9.84	9.75	11.10	16.55
								

a *n* seq (number of strains), length (median length of the sequence, nt), GC % (average GC content), PQS *n* (total number of predicted PQS with a G4Hunter score of 1.2 or more), mean PQS *f* (average PQS frequency per thousand nucleotides), min PQS *f* (lowest frequency of predicted PQS), max PQS *f* (highest frequency of predicted PQS), PQS *f* per 1000 GC (PQS frequency per 1000 G or C nucleotides).

As HDV exhibits substantial genetic variability with eight genotype groups,^14^ we divided HDV genomes according to this classification. A comparison of HDV divided by genomes is shown in Figure 2. The mean PQS frequencies varied from 5.62 for HDV genomes from *genotype* 3 to 9.84 in *genotype* 8. *Genotype* 3 was more prevalent in South America and *genotype* 4 was more prevalent in Asia, which corresponds to the lowest PQS frequencies of HBV in various continents. Similarly, *genotypes* 1 and 8, which have the highest PQS frequencies, are more prevalent in Africa. HBV genotypes differ significantly in PQS frequencies, which is in line with their classification into individual groups.

Taking into account that the highest PQS score indicates a higher probability of G4 formation, we performed additional analyses using higher G4Hunter scores (Table 2). As expected, the PQS frequency decreased with higher G4Hunter scores. This decreased number of PQS is similar to that of other genomes analyzed so far, with a significant decrease in the count of detected PQS upon the selection of higher thresholds, as found in viruses,^21,22^ bacteria,^23^ archaea,^24^ and eukaryotes.^25,26^ For most genotypes, PQS were present even in categories with G4Hunter scores above 1.8 and 2. However, the most striking decrease in PQS density was observed in HDVs with *genotype* 3, where we did not find PQS with a G4Hunter score above 1.8. Compared to other genotypes, this group also contains a relatively small number of sequenced genomes (11). On the other hand, HDV genomes within *genotype 7* follow a profile comparable to humans, where selection against stable G4 is not as strong as in *genotype* 3.

**Table 2:** PQS Frequencies per 1000 Nucleotides for Various Genotypes According to Their G4Hunter Score

		G4Hunter threshold			
genotype	*N* of sequences	>1.2	>1.5	>1.8	>2.0
1	322	8.68	3.65	1.63	1.02
2	19	7.07	4.76	2.19	0.59
3	11	5.62	1.60	0	0
4	38	6.71	2.80	1.49	0.89
5	17	6.86	3.15	0.79	0.10
6	16	8.51	4.31	1.79	0.51
7	45	7.65	3.43	1.96	1.66
8	6	9.84	3.12	2.05	1.27

Importantly, all genomes available from American samples do not exhibit any PQS with a G4Hunter score of >1.8 (Table 3). The highest PQS frequencies with G4Hunter scores greater than 1.5, 1.8, or 2.0 were all found in the HDV genomes of African origin.

**Table 3:** PQS Frequencies per 1000 Nucleotides for Various Genotypes According to Their G4Hunter Score

		G4Hunter threshold			
continent	*N* of sequences	>1.2	>1.5	>1.8	>2.0
Africa	230	8.88	3.86	1.99	1.33
Asia	186	7.55	3.31	1.38	0.75
Europe	24	9.19	3.37	1.10	0.44
North America	3	7.84	2.55	0	0
South America	9	5.56	1.70	0	0

**Figure 3:**
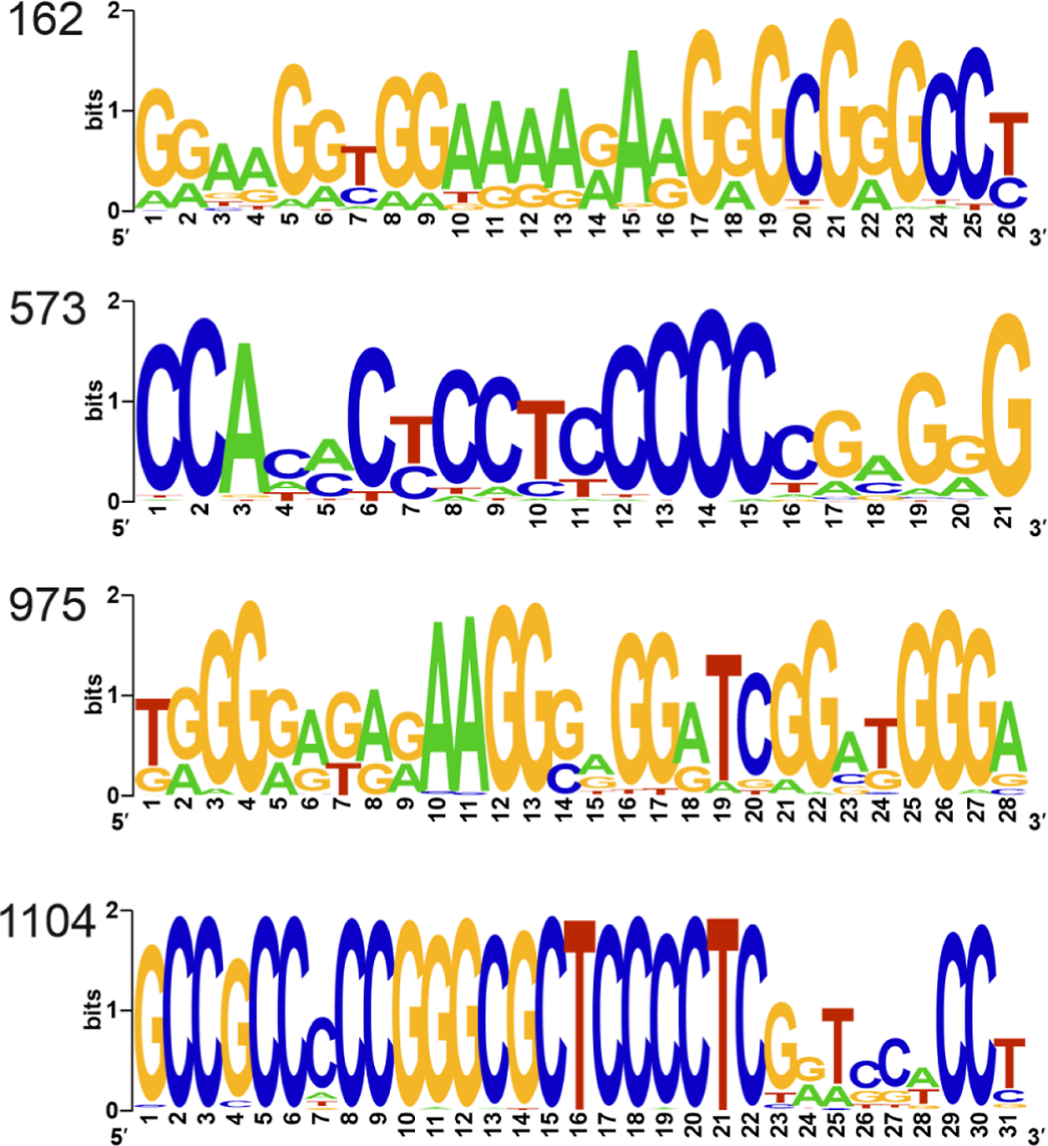
Conservation of bases in the selected G4s. LOGO representation of the consensus PQS motifs in four locations (indicated on the left) with the highest G4Hunter scores.

To check the conservation of G or C nucleotides in the PQS sequences, we used the four most frequent PQS for visualization of nucleic acid multiple sequence alignment by LOGO sequences. Selected PQSs were placed at starting bases 162, 573, 975, and 1104 according to the D01075.1 genome. Compared with HBV (a DNA virus), sequences within HDV (an RNA virus) genomes are less conserved. However, as shown in Figure 3, the bases potentially involved in G4 formation (G or C when the complementary strand is concerned) demonstrate greater conservation than other bases, as evidenced in Figure 3 (the taller the letter, the higher the conservation). The conservation of the nucleotides involved in G4 formation supports their biological importance.

**Figure 4:**
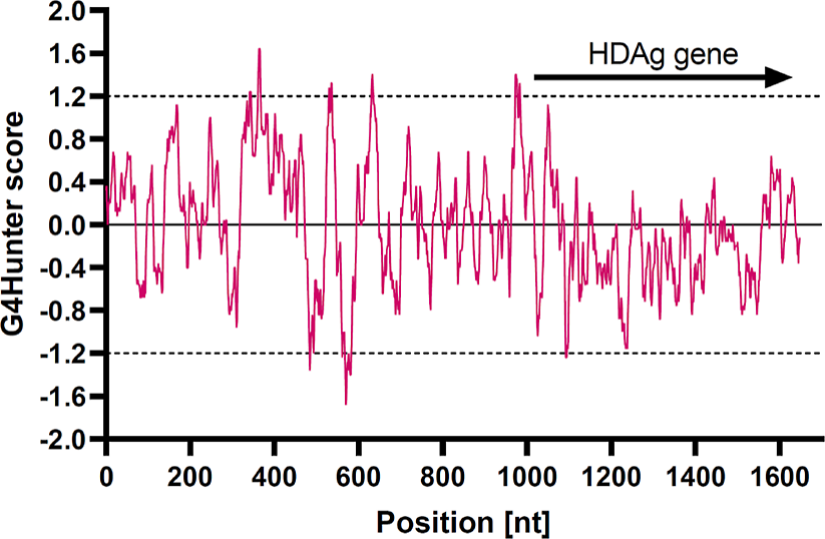
PQSs location within the reference HDV genome (NC_076103.1). The regions identified as PQS by G4Hunter, have either positive scores exceeding 1.2 (above the dashed line) or negative scores (below −1.2, PQS in the complementary antigenome sequence). The coding region is shown by arrow.

To gain insight into the potential roles of quadruplexes in HDV, we determined the specific locations of PQS within its genome. Genomic locations discussed in this study were classified according to the annotation definitions provided by the NCBI database. The frequency of PQS in regions located within the annotated features was analyzed. Through a comprehensive analysis of all HDV genomes, we present the distribution of PQS in the HDV reference genome in Figure 4. The majority of PQS were found in the noncoding regions, located between nucleotide positions 300 and 1000. PQS enrichment was also observed in the repeat regions (Supporting Information 02). Interestingly, a single PQS sequence is found in the HDV coding sequence.

Although G4 structures are well-documented in DNA and have been extensively studied for their potential roles in gene regulation, their presence in viral RNA has gained attention in recent years.^27,28^ Furthermore, RNA GG4s are suggested to constitute a good target for antiviral therapy.^29,30^ Previously, G4s in lentiviruses were studied, and their role in HIV infection was demonstrated.^31,32^

In contrast, PQS tend to be counterselected in RNA viruses. For example, the number of G4s in the SARS-CoV-2 genome is very limited.^21,33^ Still, G4 stabilization was used to inhibit viral infection,^34,35^ perhaps by acting on cellular RNA G4s.^36^ The high PQS propensity found in HDV, an RNA virus (8.25 per 1000 nucleotides), is therefore striking, when compared to the frequencies observed in the HBV (DNA virus) and human genomes (1.69 and 1.94, respectively),^20^ supporting potential roles of G4s in the life cycle of HDV.

Non-B DNA structures have been shown to play an important role in viruses due to their effects on mutability, translation, and replication.^33,37^ Besides G4s, inverted repeats forming hairpins and cruciform have been described, as well as triplexes in homopurine-homopyrimidine sequences.^38−40^ G4s may modulate viral gene expression,^41^ the host immune response, the packaging of viral RNA, genome stability (since G4 structures can help stabilize certain regions of viral RNA, protecting them from enzymatic degradation). All of these features may lead to positive selection.

On the other hand, G4s pose problems for the replication and translation machineries, especially when fast turnover is sought. Furthermore, small-molecule chaperones facilitate RNA G4 folding.^42^ Therefore, the high frequency of PQS in the HDV genome can serve as a selective target to prevent its replication and spreading. Finally, RNA PQS may serve as sites for RNA modification to occur. Two members of the adenosine deaminase that act on RNA (ADAR) family, ADAR1 and ADAR2, catalyze the deamination of adenosine to inosine in perfect and imperfect duplex RNA.^43^ Burrows and colleagues reanalyzed previous sequencing data that identified m6A installation within the loops of two-tetrad PQSs in the RNA genomes of the Zika, HIV, hepatitis B, and SV40 viruses.^44^ Even if HDV was not specifically investigated, these observations open new perspectives on the interplay between mRNA modifications and secondary structures, providing new incentives to study them in infected cells.

## Supporting Information Available

List of all 6675 PQS found in HDV genomes, statistical data and their evaluation (excel file), and PQS frequencies according to the annotation and other annotated locations (ZIP)

## Abbreviations

HDV: hepatitis delta virus
PQS: potential quadruplex-forming sequences
G4: G-quadruplex
