# Supplementary material for: Abundance of G-Quadruplex Forming Sequences in the Hepatitis Delta Virus Genomes
Source: ACS Omega. 2024 Jan 9;9(3):4096–101. doi: 10.1021/acsomega.3c09288 (PMC10809645; doi:10.1021/acsomega.3c09288)
Supplement: Supplementary file 1 — ao3c09288_si_001.zip [file ao3c09288_si_001.zip › SM_02.pdf]

Abundance of G-quadruplex forming sequences in the Hepatitis Delta virus genomes

Václav Brázda [a,b*], Natália Valková [a], Michaela Dobrovolná [a,b], Jean-Louis Mergny [a,c]

[a] Institute of Biophysics of the Czech Academy of Sciences Královopolská 135. 621 00, Brno, Czech Republic.
[b] Faculty of Chemistry, Brno University of Technology, Purkyňova 118, 61200, Brno, Czech Republic.
[c] Laboratoire d'Optique et Biosciences, Ecole Polytechnique, CNRS, INSERM, Institut Polytechnique de Paris, 91120 Palaiseau, France.

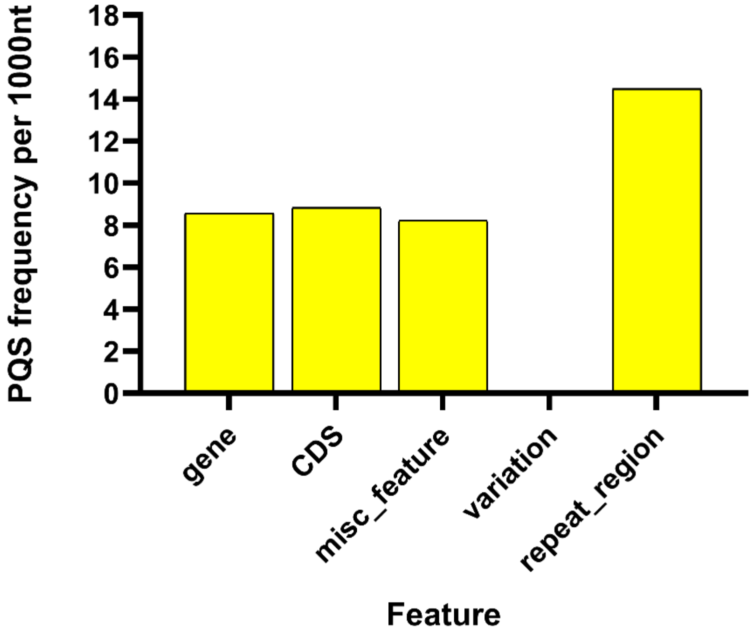

Supplementary material 02: PQS frequency in HDV genome annotated parts
